# Some Commonly Used Brominated Flame Retardants Cause Ca^2+^-ATPase Inhibition, Beta-Amyloid Peptide Release and Apoptosis in SH-SY5Y Neuronal Cells

**DOI:** 10.1371/journal.pone.0033059

**Published:** 2012-04-02

**Authors:** Fawaz Al-Mousa, Francesco Michelangeli

**Affiliations:** School of Biosciences, University Of Birmingham, Edgbaston, Birmingham, United Kingdom; Massachusetts Eye & Ear Infirmary, Harvard Medical School, United State of America

## Abstract

Brominated flame retardants (BFRs) are chemicals commonly used to reduce the flammability of consumer products and are considered pollutants since they have become widely dispersed throughout the environment and have also been shown to bio-accumulate within animals and man. This study investigated the cytotoxicity of some of the most commonly used groups of BFRs on SH-SY5Y human neuroblastoma cells. The results showed that of the BFRs tested, hexabromocyclododecane (HBCD), tetrabromobisphenol-A (TBBPA) and decabromodiphenyl ether (DBPE), all are cytotoxic at low micromolar concentrations (LC_50_ being 2.7±0.7µM, 15±4µM and 28±7µM, respectively). They induced cell death, at least in part, by apoptosis through activation of caspases. They also increased intracellular [Ca^2+^] levels and reactive-oxygen-species within these neuronal cells. Furthermore, these BFRs also caused rapid depolarization of the mitochondria and cytochrome *c* release in these neuronal cells. Elevated intracellular [Ca^2+^] levels appear to occur through a mechanism involving microsomal Ca^2+^-ATPase inhibition and this maybe responsible for Ca^2+^-induced mitochondrial dysfunction. In addition, µM levels of these BFRs caused β-amyloid peptide (Aβ-42) processing and release from these cells with a few hours of exposure. These results therefore shows that these pollutants are both neurotoxic and amyloidogenic *in-vitro.*

## Introduction

Brominated flame retardants (BFRs) are chemicals commonly used to reduce the flammability of a variety of consumer products such as electrical goods and foam furnishings [1]. These chemicals are widely dispersed and slowly degraded within the environment and are therefore referred to as persistent organic pollutants (POPs) [1], [2]. Several studies have suggested that some BFRs such as the polybrominated diphenyl ethers (PBDEs) are able to cross the blood-brain barrier [3] and cause neurological disorders in developing animal models by affecting learning, memory and motor functions [4]–[6]. Therefore, these compounds could also potentially pose a risk to human health [7]. However, few studies have been undertaken to investigate whether the currently used BFRs such as tetrabromobisphenol-A (TBBPA) and hexabromocyclododecane (HBCD) also have neurotoxic effects. Therefore as an initial step towards investigating this, detailed in-vitro studies would need to be undertaken.

It is now well documented that many of these chemicals do bio-accumulate within animals including man [7]. Analysis of human blood samples has detected relatively high concentrations of some of these pollutants. For instance DBPE (BDE-209) has been shown to reach levels of 3100ng/g blood lipid (equivalent to 3µmol/l), in some blood samples from workers employed in electronic dismantling facilities [8]. Another study showed HBCD to be as high as 850ng/g blood lipid (equivalent to 1.3µmol/l) in humans working in polystyrene manufacture [9]. It is unknown to what levels these chemicals can reach in human brains, however, animal studies have shown that they are able to cross the blood brain barrier [3] and given their high lipophilicity [10] accumulation in the brain is likely.

Most attention has so far focussed on the potential neurodevelopmental effects of BFRs [4]–[6] rather than their presence as potential risk factors associated with neurodegenerative diseases. In this study we therefore focus on the potential cellular disrupting effects of some commonly used BFRs by assessing their affects on the viability of SH-SY5Y human neuronal cells, investigating their mechanisms by which they cause neuronal cell death, and their ability to produce β-amyloid peptide (Aβ), a key pathological biomarker for neuro-degeneration such as in Alzheimer’s disease.

## Materials and Methods

### Chemicals

Tetrabromobisphenol-A (TBBPA) (purity of 97%) (Acros Organics, UK), Hexabromocyclododecane (HBCD) (purity of 95%), and Decabromodiphenyl ether (DBPE) (purity of 97%) were purchased from Sigma-Aldrich, The amino-methylcoumarin (AMC) fluorogenic caspase substrate Ac-DEVD-AMC (caspase-3/7) was purchased from Alexis Biochemicals (UK). The caspase inhibitor Z-IETD-FMK [11], [12] was purchased from Merck Chemicals Ltd (UK). Dulbecco’s Modified Eagle’s medium (DMEM) and foetal bovine serum (FBS) were purchased from Lonza (UK).

### Cell Culture

The SH-SY5Y neuroblastoma cells (from ATCC) were grown in Dulbecco’s modified Eagle’s medium (DMEM) supplemented with 2 mM L-glutamine,1% penicillin (20 units/ml), streptomycin (20 mg/ml), and containing 10% (vol/vol) heat-inactivated foetal bovine serum (FBS). Cells were maintained at 37°C in a saturated humidity atmosphere containing 95% air and 5% CO_2_.

### Cell Viability Assay

Cells were seeded in 24-well cell culture plates and allowed to grow at 37°C until about 70% confluency was reached (typically, 4×10^4^ cells/well). Treatment with the chemicals was undertaken in the culture medium with DMEM (high glucose / without phenol red or FBS). Stock solutions of the chemicals were prepared in dimethyl sulfoxide (DMSO) and no more than 1% v/v was added to the cells. The cells were exposed to varying concentrations of the chemicals for 24 h and cell viability was determined by MTT assay as described in [13], [14]. Cell viability with HBCD was also assessed by propidium iodide staining and FACs analysis as described in [15]. In some cell viability assays the caspase inhibitor, Z-IETD-FMK (40µM) was used.

### Caspase Activity

Caspase-3/7 activity in cell lysates were assayed using the fluorogenic substrate Ac-DEVD-AMC. SH-SY5Y cells (5×10^5^ cells) were incubated in the presence of the chemicals for typically 12h and then lysed in 500 µl of lysis buffer (10 mM Tris–HCl, pH 7.5, 130 mM NaCl, 1% Triton-X- 100, 10 mM Na_4_P_2_O_7_, and 10 mM Na_2_HPO_4_), on ice. To 1ml of protease buffer (20 mM HEPES, 10% glycerol and 2 mM dithiothreitol, pH7.5) was added 20 µM of substrate (final concentration) and 100 µl of the cell lysate, and the mixture was incubated for 1 h at 37°C. The fluorescence (due to release of AMC from the peptide) was measured with a spectrofluorometer set at the excitation wavelength of 380 nm and emission wavelength of 460 nm and comparing the values to a standard curve using 7-amino-4- methylcoumarin.

### Cytochrome c Release Assay

Detection of cytochrome c in mitochondrial and cytosolic protein extracts was undertaken by immunoblotting. SH-SY5Y cells (5×10^5^ were incubated in the presence of compounds (dissolved in DMSO) in DMEM culture medium for 12 h. The cells were suspended in lysis buffer (20 mM HEPES, pH 7.5, containing 10 mM KCl, 1.5 mM MgCl_2_, 1 mM EDTA, 1 mM EGTA, 1 mM DTT, and 1 mM phenylmethylsulfonyl fluoride), and disrupted using a Dounce homogenizer. The cell lysates were centrifuged at 10,000*g* for 10 min and the supernatants were further centrifuged at 100,000*g* for 30 min. Proteins in the final supernatant (cytosolic fraction) and first centrifuged pellet (incorporating the mitochondrial fraction) were separated by 15% SDS-PAGE followed by electro blotting onto nitrocellulose membranes. After blocking and washing, the membrane was then probed with an anti-cytochrome c antibody (C-20; Santa Cruz Biotechnology, Inc), at a dilution of 1:500 for 1 h. and cross-reactivity was detected using secondary antibodies as described in [16].

### Measurement of Mitochondrial Membrane Potential (MMP)

Mitochondrial membrane potential (MMP) in SH-SY5Y cells were monitored using the fluorescent dye Rhodamine123 (Rh123) as described in [13], [14].

### Detection of Reactive Oxygen Species

Reactive oxygen species (ROS) formation was measured by using the fluorescent probe 2',7'-dichlorofluorescein diacetate (DCFH-DA) which forms 2',7'-dichlorofluorecein (DCF) when oxidized by ROS. SH-SY5Y cells were cultured to 70% confluency in 12-well plates and treated with the compounds for 24 h, and then subsequently washed with PBS and loaded with 40 µM DCFH-DA (added in DMSO) at 37°C with 5% CO_2_ and constant humidity for 30 min. At the end of the incubation, the cells were washed with PBS. 100 µl NaOH (1 M) was added to extract the fluorescent product from the cells. The fluorescent intensity of the cell extracts were measured with a Perkin Elmer LS-50B spectrofluorimeter (excitation 485 nm and emission 530 nm). ROS formation was expressed as the amount of DCF formed using a DCF standard curve and then compared to control cell values.

### Fluorescence Measurement of Changes in Intracellular [Ca^2+^]

SH-SY5Y cells were allowed to grow to 70% confluency on gelatin-coated coverslips. The coverslips were incubated in sodium hydrogen carbonate-supplemented HBSS (pH 7.2), which contained 0.08 µM sulfinpyrazone, 1% bovine serum albumin, 0.025% pluronic acid and 10 µM Fluo-3-acetoxymethyl ester (Fluo-3 AM) for 50 min. This solution was then removed, replaced with fresh HBSS containing 0.08 µM sulfinpyrazone, and incubated for an additional 20 min. Each coverslip was then moved into a 35 mm plastic petri dish containing fresh HBSS (2 ml final volume), placed onto a heated microscope stage maintained at 35°C and cells were observed with a Nikon TS100F microscope in epi-fluorescence mode. The microscope was fitted with an FITC filter cube so that fluo-3 fluorescence could be monitored. Recordings of the cells, viewed at about 200x magnification, were taken using an Astrovid StellaCam^3^ connected to a Hauppauge USB TV live video capture device for viewing on a PC. Win TV (Hauppauge; version 1.4) was used to record fluorescence images of the cells at a frame rate of 1 frame/s. Recordings were initiated about 60s before the chemicals were added, which allowed the initial un-stimulated fluorescence intensity (F_o_) to be determined. All compounds were dissolved in DMSO cells were exposed to ≤ 1% DMSO in experiments (this maximum concentration had no effect on the Fluo-3 fluorescence intensity of cells when added alone). In the case of HBCD, 2-hydroxypropyl-β-cyclodextrin (150mg/ml) was also added to improve aqueous solubility. Each series of images were analysed using Image J software (version 1.32j; National Institutes of Health USA). For each recording, the analysis involved the measurement of the mean intensity /cell area for a number of cells. After corrections for background fluorescence and photo-bleaching were made, these values were then converted into ratios of fluorescence intensities with respect to unstimulated fluorescence intensity (F/F_o_) for each cell.

### Ca^2+^ATPase Activity Measurements

Microsomal membranes were isolated from SH-SY5Y cells by homogenisation and differential centrifugation as previously described in [16]. Ca^2+^-ATPase activity (Ca^2+^-dependent ATP hydrolysis) from the SH-SY5Y cells was measured using the phosphate liberation assay as described in [14], [16].

### Aβ-42 –ELISA

Aβ 1-42 level was determined by using the BetaMark x-42 ELISA kit (Covance). Cells (5×10^6^) were exposed to HBCD, TBBPA and DBPE for up to 12h. After the desired incubation times, cell culture supernatants were removed and centrifuged to remove any cellular debris. Proteinaceous material from the supernatant was precipitated with 10% trichloroacetic acid (TCA) for 15 min at 4°C followed by centrifugation at 21,000g. The resultant pellets were resuspended in a buffer containing 150 mM Tris- HCl buffer (pH 7.5) containing 150 mM NaCl, 1% Nonidet P-40, 0.1% sodium dodecyl sulfate, 2 m*M* EDTA, in presence of PMSF (1mM) and leupeptin (10µM) and diluted with working incubation buffer according to the manufacturer’s instructions. Aβ-42 was then detected using an antibody-amyloid-antibody (sandwich) complex and compared to values gained using a known amount of Aβ-42 peptide as a standard. The experiments were performed in triplicate.

### Statistical Analysis

All data values are expressed as the mean and include standard deviations (S.D). Statistical significance (*p*) of selected data, compared to controls, were analyzed using either Student’s *t*-test or the ANOVA multiple comparison test.

## Results

### Effects of Some BFRs on SH-SY5Y Neuronal Cell Viability

**Figure 1:**
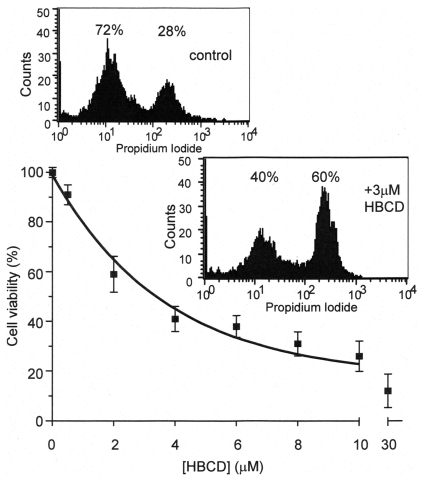
The effects of hexabromocyclododecane (HBCD) on SH-SY5Y cell viability. The SH-SY5Y cells were incubated with HBCD over a range of 0 to 30µM concentration for a period of 24 hours. The cell viability was then monitored using the MTT assay. The data points are the mean±S.D. of 4 determinations. (Inset) SH-SY5Y cells were incubated in the presence or absence of 3µM HBCD for 24 hours, then labelled with propidium iodide and counted using a fluorescence-activated cell counter. The percentage values relate to the proportion of viable (left hand value) and non-viable (right hand value) cells and were typical of 3 separate experiments.

Using the MTT cell viability assay it can be deduced that of the 3 chemicals tested (over a 24 hour exposure), HBCD was the most potent at inducing cell death in the SH-SY5Y neuroblastoma cells (figure1). The LC_50_ for HBCD was calculated to be 2.7±0.7µM which was similar to the value observed for HBCD in cerebellar granule cells [17] and may indicate that a range of neuronal cell types are similarly affected by this chemical. HBCD (3µM) also caused an approximately 2-fold increase in the number of non-viable cells as determined by propidium iodide stained cells and FACS analysis (Figure 1, inset). The LC_50_ for TBBPA and DBPE using the MTT assay were determined to be 15±4 µM and 28±7 µM, respectively.

### Involvement of Caspases

**Figure 2:**
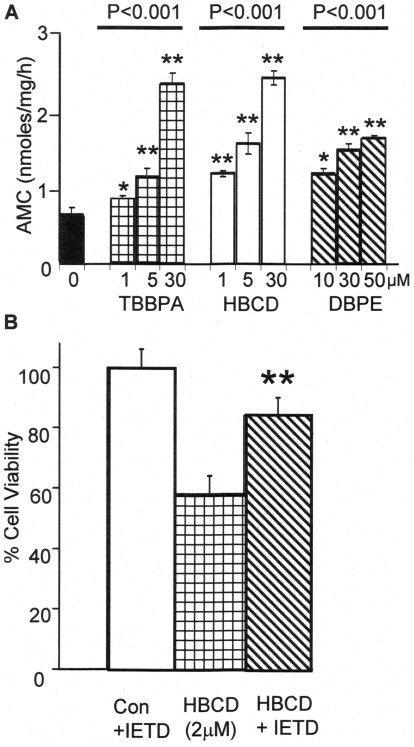
BFRs increase the activities of caspases in SH-SY5Y cells. (A) shows the effects of BFRs (TBBPA, HBCD and DBDE) at a range of concentrations, increase caspase 3/7 activity (using Ac-DEVD-AMC as the substrate) in SH-SY5Y cells when treated for 12 hours. (B) Shows the % cell viability of SH-SY5Y cells treated with or without 2µM HBCD and cells pre-treated with caspase inhibitor Z-IETD-FMK (40µM). (Con) are cells pre-treated with Z-IETD-FMK but without HBCD. The values presented are the means±SD of 4 determinations (* and ** indicates the probability of the values being significant form control data as determined by t-test with the values having P≤0.05 and P≤0.01, respectively). For comparisons of multiple data sets, ANOVA tests of the probability are given by the P value above the data set lines.

It is unclear whether apoptosis is involved in the cytotoxicity by some BFRs [17], [18]. Apoptosis is executed by caspase 3/7, activated through two major pathways, intrinsically (involving the mitochondria and abnormally elevated intracellular [Ca^2+^]) or extrinsically (through death receptors) [19]. Figure 2A shows the effects of different concentrations of HBCD, TBBPA, and DBPE on caspase-3/7 activity after the cells were exposed to these chemicals for 12 hours. As can be seen, all three BFRs caused a significant dose-dependent increase in caspase 3/7 activity over the concentration ranges tested. The most potent were HBCD and TBBPA, both showing elevated levels of caspase 3/7 activity compared to control cells at a 1 to 5µM concentration range. DBPE at 10µM and above also increased the caspase activity. There was also a rapid onset of caspase activation since within 4 hours post exposure with HBCD (5µM), caspase 3/7 activity increased significantly from 0.7±0.1 to 1.2±0.1 nmoles AMC produced/mg/h. In order to determine whether cell death was through caspases-dependent apoptosis, the level of cell death caused by of HBCD (2µM) in the presence of the caspase inhibitor Z-IETD-FMK was measured (figure 2B). The inhibitor afforded significant protection from cell death, indicating that caspases were, at least in part, involved in this process.

### Involvement of the Mitochondria

**Figure 3:**
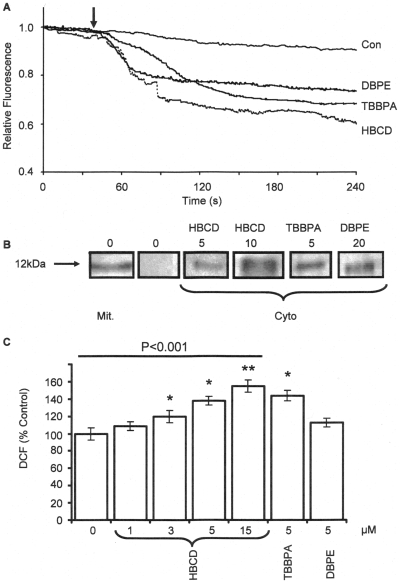
Effects of BFRs on mitochondrial depolarisation, cytochrome-c release and ROS generation in SH-SY5Y cells. (A) Shows the effects of DBPE (10μM), TBBPA (10μM), and HBCD (10μM) on mitochondrial membrane depolarisation of SH-SY5Y cells loaded with rhodamine123. The traces are the averages of 10 individual cells, within the field-of-view and are typical of 3 replicate experiments. The arrow indicates the time point at which the BFRs were added to the cells. (B) Shows the effects of SH-SY5Y cells (5x10^5^) treated with HBCD (5, 10μM), TBBPA (5μM) and DBPE (20μM) for 12 hours. The cells were then homogenised and centrifuged to obtain the mitochondrial and cytosolic fractions. The fractions were the subjected to SDS-PAGE and immuno-stained with an anti-cytochrome c antibody. The blots were typical of two replicate experiments. (C) Shows the effects of HBCD (1 to15 μM), TBBPA (5μM) and DBPE (5μM) on ROS production as measured by the formation of dichlorofluorescein (DCF). The cells were treated with the chemicals for 24 hours and then incubated with DCFH-DA for 30 minutes before lysis with NaOH and measuring the DCF fluorescence. The data represents the mean±SD of 4 determinations (* and ** indicates the probability of the values being significant form control data of P≤0.05 and P≤0.01, respectively, using the student’s t-test). For multiple comparison data sets, ANOVA tests were performed (highlighted by the line) and the P values are given.

In order to determine whether mitochondria are involved in the cytotoxicity caused by BFRs a number of mitochondrial processes were studied. Figure 3A shows that DBPE, TBBPA and HBCD (all applied to the cells at 10µM), induced mitochondrial depolarisation of the cells within a few seconds of addition, as assessed by a rapid decrease in rhodamine123 fluorescence of the cells. Each trace was the average of the fluorescence change observed for 10 cells analyzed within the field-of-view through the fluorescence microscope (about 1/3 of the total cells) and was typical of 3 replicates. When no compound was added only a slow rate of fluorescence decrease was observed, due to photo-bleaching [14].

Figure 3B shows the effects of cytochrome *c* release as assessed by immuno-blotting analysis of the mitochondrial fractions and cytosolic fractions obtained from SH-SY5Y cells following treatment with the BFRs for 12 h. In control cells all the cytochrome c was detected in the mitochondrial fraction with none detected in the cytosolic fraction. Cytochrome *c*, however, appeared in the cytosol of SH-SY5Y cells after treatment with 5 & 10 µM HBCD, 10 µM TBBPA and 20 µM DBPE.

One consequence of compromised mitochondria is oxidative stress which generates potentially damaging reactive oxygen species (ROS). Figure 3C shows the generation of ROS as monitored by the oxidation of DCF when the cells were exposed to the chemicals for 24 hours prior to measurements. The figure shows that HBCD caused a dose-dependent increase in DCF fluorescence compared to control cells, with significant levels of detection above 3µM HBCD. TBBPA also showed significant levels of ROS increase compared to control cells.

### Changes of Intracellular [Ca^2+^] Levels & Ca^2+^ ATPase Activity in SH-SY5Y Cells

**Figure 4:**
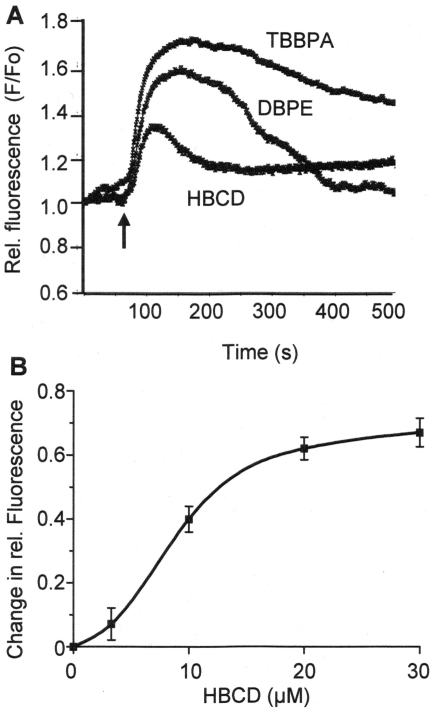
BFRs cause transient increases in intracellular [Ca^2+^] levels in SH-SY5Y cells. (A) Shows the effects of TBBPA (20μM), DBPE (20μM) and HBCD (10μM) on the transient elevation of intracellular [Ca^2+^] levels in SH-SY5Y cells as monitored using Fluo3-AM. The arrow indicates the point of addition of the chemicals. The changes in relative fluorescence intensities (F/Fo) are indicative to changes in intracellular [Ca^2+^] concentrations and the traces are the averages of 10 individual cells with the field-of-view. The traces are typical of 3 replicate experiments. (B) Shows the dose-dependent effects of HBCD on the maximal (peak) elevation in intracellular [Ca^2+^] levels (presented as change in relative fluorescence). The data points are the mean±SD of 3 determinations at each concentration.

Previous studies in this laboratory with TBBPA in testicular cells have shown that they cause elevation of intracellular [Ca^2+^] levels in these cells by inhibiting SERCA Ca^2+^ pumps [14], [20]. From the literature it is uncertain whether BFRs can also cause changes in [Ca^2+^] levels in neuronal cells [17]. Figure 4A shows the effects of HBCD, TBBPA and DBPE on intracellular [Ca^2+^] levels using Fluo-3 AM loaded SH-SY5Y cells. SH-SY5Y cells were exposed to HBCD at a concentration of 10µM, while TBBPA and DBPE were used at 20µM. The figure shows traces of the relative fluorescence changes (indicative of changes in [Ca^2+^]i) averaged for 10 individual cells (typical of 3 repeats) viewed, using the fluorescence microscope, the profiles of which were typical of the majority of cells observed. The arrow indicates the addition of the BFRs after around 60s from the start of the recording. The rise in [Ca^2+^]i measured as an increase in relative fluorescence attained maximal levels within about 1min of the addition of the chemicals. These elevations in intracellular [Ca^2+^] levels were transient in nature and returned back towards un-stimulated levels over a 5 min period. Figure 4B shows the dose-dependent effects of HBCD concentration on peak intracellular [Ca^2+^] levels within SH-SY5Y cells as monitored by the maximum level in fluorescence attained. The concentration required to cause 50% of the maximal increase in intracellular [Ca^2+^] levels was calculated to be 9±1 µM for HBCD.

**Figure 5:**
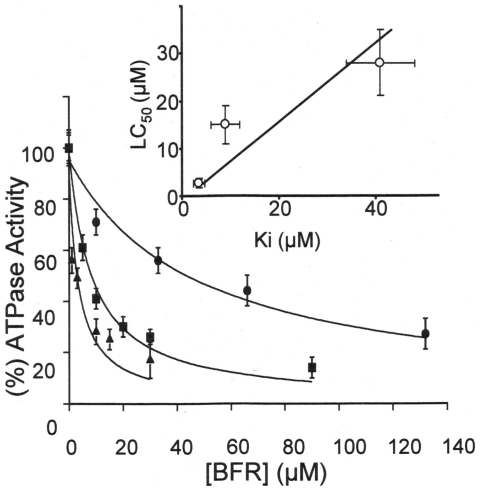
BFRs cause SH-SY5Y cell microsomal Ca^2+^-ATPase inhibition. The figure shows the effects of HBCD (▴), TBBPA (▪) and DBPE (•) on the Ca^2+^-ATPase activity from microsomes derived from SH-SY5Y cells measured at pH7.0 and 37°C. The data is presented as the mean±SD (n = 4). Inset shows the correlation between the inhibition constants for the BFRs derived from the data in figure 5 with their LC_50_ values for cell death. From left to right, the points relate to data for HBCD, TBBPA and DBPE, respectively.

The effects of these BFRs were then assessed on microsomal membranes isolated from SH-SY5Y cells in order to determine whether they directly inhibited SERCA Ca^2+^-ATPase activity. Figure 5 shows that all 3 BFRs tested inhibited the microsomal Ca^2+^-ATPase activity from these cells. HBCD appeared to be the most potent with an inhibition constant Ki of 3.5±1.2µM. The inhibition constants determined for TBBPA and DBPE were 9±2µM and 41±7µM, respectively. Figure 5(inset) also shows that for these BFRs there is a positive trend between their potency of Ca^2+^-ATPase inhibition (Ki) and their potency at inducing cell death (LC_50_).

### BFRs Cause Secretion of β-amyloid Peptide from SH-SY5Y Cells

**Figure 6:**
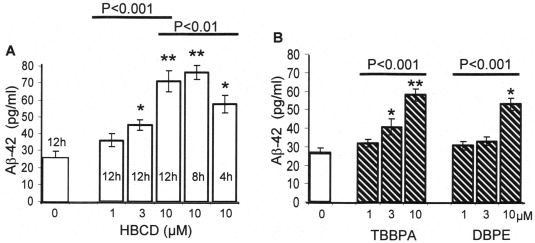
BFRs cause Aβ-42 peptide secretion in SH-SY5Y cells. (A) Shows the effects of HBCD (1 to 10µM) on the production of Aβ-42 peptide secreted into the medium and detected by a sandwich ELISA. The figure also shows how HBCD increases the production of Aβ-42 peptide in a time-dependent manner. (B) Shows the effects of Aβ-42 production in cells treated with TBBPA and DBPE (1 to10µM) for 12 hours. The data represents the mean±SD of 3 determinations (* and ** indicates the probability of the values being significant form control data using student’s t-test of P≤0.05 and P≤0.01, respectively). ANOVA multiple comparison tests were also performed on multiple data sets (highlighted by the line). P values are given.

There is evidence to link dis-regulation of Ca^2+^ homeostasis within neuronal cells and the initiation of neuronal cell degeneration and cell death which can occur in diseases such as in Alzheimer’s disease (AD) [21], [22]. One of the key molecular factors in neuronal degeneration is the formation of β-Amyloid plaques which are known to be neurotoxic [23], [24]. β-amyloid plaques are formed from the aggregation of soluble β-Amyloid peptides which have been cleaved from the plasma membrane bound amyloid precursor protein (APP) by the action of γ and β secretase enzymes [23]. In order to determine whether SH-SY5Y cells treated with BFRs induce the formation and cellular release of β-amyloid peptide (Aβ), the extracellular fluid was analysed and quantified by sandwich ELISA using a specific β-amyloid-42 antibody. Several Aβ peptides can be generated, however, Aβ-42 is believed to be the most fibrillogenic and is therefore most closely associated with AD [24]. Figure 6A shows that HBCD causes an increase in the extracellular amount of Aβ-42 in both a time-dependent and dose-dependent fashion. Significant levels of Aβ-42 peptide were detected, compared to control, in cells exposed for only 4 hours with HBCD. Figure 6B also shows that concentrations of 3 to 10µM of TBBPA and DBPE, when added to cells for 12 hours also significantly increased the levels of Aβ-42 above those of control cells.

## Discussion

Over recent years much attention has been given to the potentially neurotoxic effects of BFRs particularly in impairment of neurological development and function [4]-[6]. These affects may be due to direct interactions of these compounds with neuronal cells since animal studies have shown that some BFRs are able to cross the blood-brain barrier and accumulate within brain tissue [3]. Several rodent studies have also shown that gestational or neonatal exposure to environmentally relevant concentrations of BFRs altered spontaneous behaviour and disrupted habituation in the adult animal and had profound effects on learning and memory [4]–[6].

*In-vitro* studies have shown that a variety of the BFRs are cytotoxic and can affect a range of neuronal cell functions, such as cell signalling processes, neurotransmission and cell death [17], [25], [26]. However, little is known of the molecular mechanisms of these effects.

This study has therefore undertaken an investigation on a number of BFRs to determine their effects on cultured SH-SY5Y human neuronal cells. The results presented here show that these BFRs (and particularly HBCD) are highly cytotoxic, causing cell death via caspase-dependent apoptosis. The most likely mechanism is that these BFRs activate the intrinsic apoptotic pathway through exaggerated temporal increases of intracellular [Ca^2+^] levels, caused by inhibition of the microsomal Ca^2+^-ATPase (SERCA), leading to mitochondria dysfunction which is manifested as both mitochondrial membrane depolarization and cytochrome *c* release [13], [14], [27].

There is substantial evidence to indicate that molecular lesions associated with AD are caused by dis-regulation of Ca^2+^ signalling and mitochondrial function [21], [22], [28]. Some of this evidence has been generated using cells expressing presenilins (which have been shown to be mutated in certain forms of genetically induced familial forms of AD) [21], [29], [30]. Presenilins form part of the γ-secretase complex which specifically cleaves the C-99 fragment of APP (∼12kDa) to Aβ peptide (∼4kDa). Mutant presenillins have also been shown to affect store-operated Ca^2+^ entry into cells [31], increase the activity and / or expression of intracellular Ca^2+^ channels such as the Ryanodine receptor and InsP_3_ receptor [29], [32]–[34], and modulate the function of SERCA Ca^2+^ ATPase [35]. Furthermore, it is believed that Aβ peptides form oligomers within the plasma membrane of neurons which then cause excessive Ca^2+^ influx into the cells [36]. In fact one popular current idea is that of the ‘ER Ca^2+^ overload hypothesis’, whereby ER and other intracellular Ca^2+^ stores become overfilled with Ca^2+^, leading to exaggerated increases in cytosolic [Ca^2+^] levels [21], [22], [36]. Alterations in cytosolic [Ca^2+^] levels can also be induced by modulation of Ca^2+^ re-uptake due to changes in Ca^2+^-ATPase expression and or indeed Ca^2+^-ATPase inhibition [37], [38]. Although the effects of excess cytosolic Ca^2+^ can initially be subtle and have negligible pathological effects, with increasing age (or chronic low level toxicological exposure) the Ca^2+^ homeostatic machinery is known to become less effective [39], ultimately leading to Ca^2+^- induced neuronal cell death.

The observation that micromolar concentrations of HBCD and TBBPA are able to generate measurable levels of ROS may have implications for the role of free radical damage to cellular components such proteins, membranes and DNA. One recent study using tetrabromodiphenyl ether (PBDE-47) on hippocampal neuronal cells showed clear evidence of DNA damage at 2µM using single cell gel electrophoresis ‘comet’ assay, even though an increase in cell death was not detectable at this concentration [40]. An accumulation of DNA damage over prolonged time periods caused by low levels of exposure to these types of chemicals could ultimately also lead to premature neuronal cell death.

We have previously shown that TBBPA is a potent inhibitor of SERCA Ca^2+^ pumps in skeletal muscle and testicular cells [14], [20], [37]. It now appears that other BFRs also can inhibit SERCA Ca^2+^ATPases in neuronal cells. Since a correlation exists between Ca^2+^-ATPase inhibition and cell viability for these BFRs, we postulate that some of these BFRs cause neurotoxicity by prolonged / exaggerated increases in cytosolic [Ca^2+^] levels due modulation of these Ca^2+^ transporters. There is now an increasing body of evidence to suggest that abnormally elevated cytosolic [Ca^2+^] levels induce a number of potentially neuro-pathological effects, *in-vitro* [21], [22], [28], [36], [41]. For instance thapsigargin (a potent SERCA inhibitor) which increases intracellular [Ca^2+^] levels has been shown to also increase the expression levels of APP in some cell types [42], and thapsigargin and other Ca^2+^ elevating agents increase levels of Aβ peptide in cells expressing APP [43]. Furthermore, elevated [Ca^2+^] increases the activity of Ca^2+^-dependent proteases (calpains) in neuronal cells, which increase the expression of β-secretase (BACE1) [44]. Activation of caspase 3 (through a Ca^2+^-dependent mechanism) also increases β-secretase activity through the degradation of GAA3 an adaptor protein involved in BACE trafficking [45]. All these Ca^2+^-dependent mechanisms are likely to cause increases in Aβ peptide levels within neuronal cells. If these processes were also to occur in the brain as well as in cultured neurones, then as Aβ peptides can also induce further Ca^2+^ influx into neighbouring cells [46], this could lead to a propagating cycle of neuronal cell death throughout extended regions of the brain.

It is clear from these in-vitro studies that low µM concentrations of some commonly used BFRs are neurotoxic and amyloidogenic to cultured neuronal cells. However, in order to determine whether these compounds might have implications toward neurodegenerative diseases, animal studies would need to be undertaken with a view to examining whether pathological signs of neuro-degeneration are indeed observed after chronic low dose exposures.
